# Path coefficient analysis unraveled nutrient factors directly impacted the textural characteristics of cooked whole-grain purple rice

**DOI:** 10.3389/fnut.2024.1490404

**Published:** 2024-10-29

**Authors:** Ekawat Chaichoompu, Siriluck Wattanavanitchakorn, Rungtiva Wansuksri, Wintai Kamolsukyeunyong, Siriphat Ruengphayak, Apichart Vanavichit

**Affiliations:** ^1^Interdisciplinary Graduate Program in Genetic Engineering and Bioinformatics, Kasetsart University Chatuchak, Chatuchak, Thailand; ^2^Rice Science Center, Kasetsart University Kamphangsaen, Nakhon Pathom, Thailand; ^3^Cassava and Starch Technology Research Team, National Center for Genetic Engineering and Biotechnology, National Science and Technology Development Agency, Khlong Nueng, Thailand; ^4^Innovative Plant Biotechnology and Precision Agriculture Research Team, National Center for Genetic Engineering and Biotechnology (BIOTEC), Khlong Nueng, Thailand

**Keywords:** whole-grain pigmented rice, path coefficient analyses, dietary fiber index, amylose content, amylose/amylopectin contents, soluble and insoluble dietary fiber, hardness, adhesiveness

## Abstract

**Introduction:**

Whole-grain pigmented rice (WCP) provides many nutritional benefits compared to non-pigmented varieties. The textural quality of cooked whole-grain rice, particularly its hardness, is crucial for consumers’ preferences.

**Materials and Methods:**

We investigated the impact of multiple-grain nutrient components on textural attributes through Pearson Correlation and Path Coefficient Analyses (PCA).

**Results and Discussion:**

From correlation analysis, we found that the dietary fibre index (DFI), soluble and insoluble fibre (SDF and IDF), and amylose/amylopectin contents (AC/AP) influenced hardness (HRD) significantly. Nonetheless, the binary correlation failed to detect the impact of protein on hardness; instead, it strongly affected adhesiveness (ADH). The PCA revealed protein, AC/AP, and DFI significantly impacted HRD and ADH. Furthermore, DFI antagonised protein and AC/AP to define HRD, while AC/AP and DFI opposed the direct effects of protein on ADH. DFI’s effects on HRD were more appealing among low AC than high or waxy rice groups. Instead, the effect of protein was more appealing among waxy rice varieties. Based on PCA, rice breeders can now rely on three nutrient factors, protein, DFI, and AC/AP, to redesign whole-grain pigmented rice to achieve consumer acceptance and well-being.

## Introduction

Rice (*Oryza sativa L.*) is a staple food in most Asian countries. Consumers increasingly prefer polished rice over whole-grain rice for its texture, taste, and appearance (1). The relationship between white rice consumption and Type-2-diabetes (T2D) among the Asian population has been reported (2). Nonetheless, the consumption of whole grain pigmented rice is rapidly increasing due to its rich nutritional and bioactive compounds, mainly antioxidants. Metabolic cohort studies and meta-analysis revealed that a higher intake of whole-grain rice lowered the risk of non-communicable diseases (NCDs), including Type-2-diabetes (T2D), cardiovascular diseases (CVD), and cancers. The rich bioactive compounds in rice bran and germ, including phenolic acids, flavonoids, anthocyanin, proanthocyanidin, tocopherols, tocotrienols, *γ*-oryzanol, and phytate have remarkable biological activities on anti-inflammatory against NCDs (3–13). Whole-grain pigmented rice is the richest source of flavonoid anthocyanins contained in rice bran, including light brown (leucoanthocyanin), red (cyanidin-3-glucoside anthocyanin), and navy blue (delphinidin-3-glucoside anthocyanin), and soluble fiber, proteins, fatty acids, phytosterols, vitamins, minerals, and antioxidants (14). Therapeutic effects of high antioxidant pigmented bran involve anti-atherosclerosis, anti-cancer, neuroprotective, retinol-protective, immunomodulatory, anti-aging, and anti-obesity (14).

Cooked purple rice is the most tasty variety with distinctive aromatic characteristics, which creates a good consumer perception of the nutritional benefits of whole-grain rice. However, traditional whole-grain waxy purple rice varieties are cooked hard and stale after a leftover. Variations in the textural properties and eating quality of whole-grain rice are vital for consumers’ acceptance and perception of health benefits, particularly (15–17). The nutrient properties of endosperm starch and rice bran are fractions influencing overall textural properties in whole-grain rice. Increasing amylose and protein contents influenced cooked rice hardness and overall textural characteristics in polished rice (18). Rice starch comprises amylose (AC) and mainly amylopectin (AP), approximately >70%. AC is small and linear with slight branching, whereas AP is highly branching with a single non-reducing and multiple reducing ends. Therefore, cooked rice qualities are closely associated with AC and AP’s delicate structures and molecular interaction. The ratio between AC and AP (AC/AP) influenced gelatinisation temperature and pasting attributes of rice starch (19). Genetic engineered resistant starch affected hardness and adhesiveness (20).

Protein content negatively correlated with eating quality and textural characteristics is well-documented. Seed protein forming a net-like layer surrounding starch granules reduced swelling and water absorption of starch granules impacted pasting and textural properties, particularly chewiness and stickiness in indica and japonica rice (21–23). Therefore, high-protein rice is cooked harder, with less stickiness and elasticity than lower-protein rice (24, 25).

Plant proteins comprise seed storage proteins (SSP) and structural proteins based on their functions. There are four classes of SSPs, including alkali-soluble Glutelin, alcohol-soluble Prolamin, salt-soluble Globulin, and water-soluble Albumin (26). Glutelin constitutes more than 80% of the total endosperm protein in rice, while Prolamin, Globulin, and Albumin account for 5–10% (27, 28). The functions of structural proteins in rice seeds include metabolic enzymes, ribosomes, and hormonal regulators, but the content is minor (29). Rice SSPs are mainly localized in the aleurone layer and embryo (30). Generally, Albumin is more abundant in aleurone and glume layers but evenly distributed in rice bran, fine bran, and milled rice. Glutelin is the prominent SSP rich in rice endosperm (27, 30). Glutelin with a small amount of Globulin constitute 65% of endosperm protein in Elliptical Type II proteome (PB-II) without lamellar structures, whereas Prolamin constitutes 20–30% of endosperm protein in a Spherical Type I proteome (PB-I) with concentric lamellar structures (31, 32). Albumin is significantly correlated with palatability, while the contents of Globulin, Prolamin, and Glutelin are the opposite (33). In particular, prolamin content reduced eating quality in polished rice (34).

In addition to starch fine structure, gelatinisation temperature (GT), and protein, whole-grain dietary fiber strongly affected textural attributes (35–42). Cooked brown rice containing higher dietary fiber had higher hardness than cooked white rice, decreasing consumer acceptance (15, 43–45). Although there are reports on the relationship between an increase in the dietary fiber content and the eating quality of rice, few reports have been published on the influence of dietary fiber profiles on the textural properties of whole grain rice (46).

Our previous study exhibited a strong correlation between dietary fiber index (DFI), amylose content (AC), protein content and the textural properties of cooked whole grains, including non-pigmented and pigmented rice varieties (47). The study also revealed that the average dietary fiber and protein contents were higher in whole-grain pigmented than non-pigmented rice varieties. In this study, we further investigated which nutrient components, starch, dietary fiber and protein, influence the eating quality of whole-grain purple rice. This study focused on the cooked quality of diverged whole-grain pigmented rice from local germplasm, mutagenised lines and improved varieties. We further undertook correlation and path coefficient analysis (PCA) between the nutritional components and the textural properties of cooked whole-grain pigmented rice, with varying AC/AP, protein contents, and DFI. The outcome led us to implement new biomarkers to improve the softness and palatability while promoting the nutritional value of whole-grain pigmented rice.

## Materials and methods

### Germplasm

Thirty-six rice varieties (*Oryza sativa* L. ssp. indica) used in this study, provided by Rice Science Center, Kasetsart University, were grown in July 2022 during the rainy season. They were classified based on biochemical analysis (48, 49) and allele-specific SNP markers into waxy (0–12%), low (12–20%), and high (25–33%) AC (Supplementary Tables S1, S5). The following were varieties included in each group.

#### High-amylose rice

M1-MT2; Riceberry+3# B30-G5 (B30-G5); Riceberry+3# B30-D4 (B30-D4); Pinkaset+5#2E3 (PK + 5#2E3); Riceberry+3# B30-E4 (B30-E4); Riceberry+3# B30-D5 (B30-D5); Rainbow_Rice#01 (RBR#01); Rainbow_Rice#05 (RBR#05).

#### Low-amylose rice

Klum Hom Nin (KHN), Pinkaset+5#6E1 (PK + 5#6E1); JHN-5678 (M.15678); Pinkaset+6#16F35 (PK + 6#16F35); Riceberry+3#B29-A5 (B29-A5); Riceberry+3# B11-H5 (B11-H5); Pinkaset+6#11F09 (PK + 6#11F09); Hom Dum Sukhothai 2 (HDSKT 2); Riceberry+3#B29-G7 (B29-G7); JHN-10589 (M.210589); Pinkaset+6#3G06 (PK + 6#3G06); Riceberry+Blast 005 (RBB-005); Riceberry (RB); Jao Hom Nin (JHN); JHN-10853 (M.210853); JHN-2232 (M.22232); Riceberry+3#B11-B11 (B11-B11).

#### Waxy rice

Rainbow_Rice#02 (RBR#02); JHN-2313 (M.12313); JHN-9689 (M.29689); Klum Hom (KH); F9-11047-4-1-7-1-1-2-1 (F9); Dum Mor 37 (DM37); Klum Doi Saket (KDSK); Rainbow_Rice#04 (RBR#04); Kao Niaw Leum Pua (KNLP); Niaw Dum Chor Mai Pai (DCHMP); Niaw Mali Dum (NMLD).

Whole grain rice samples were finely ground and screened into 200 μm particle size using a speed rotor mill (Pulverisette 14, Fritsch) and stored in a refrigerator at −20°C for later use.

### DNA analysis

#### DNA extraction

Two-week-old leaf samples were used for DNA extraction using the DNeasy^®^ Plant Mini Kit (QIAGEN, Hilden, Germany). The quality and quantity of the DNA were determined using Nanodrop 8000 (Thermo Fisher Scientific, USA).

#### KASP marker development and validation

Kompetitive Allele-Specific (KASP) markers were developed based on the selected functional SNPs on amylose content (wx_5UTR_G/T; *GBSSI* gene; LOC_ Os06g04200) and gelatinisation temperature [ALK_ex8_SNP_GC/TT; *SSIIa* gene (LOC_Os06g12450)] (50). KASP assays were designed using the LGC Genomics manual.^1^ The KASP assays comprised the two allele-specific forward primers that target SNP at their 3′ ends, the forward primer (F) and a standard primer at their 5′ ends, the reverse primer (R) (Supplementary Table S1).

The KASP reaction was performed on a Hydrocycler^2^ PCR machine (LGC Genomics, UK) in accordance with the following protocol: start at 95°C for 15 min, 10 touchdown cycles (95°C for 20 s; touchdown at 65°C, − 1°C per cycle, 25 s) and then 26 cycles of amplification (95°C for 10 s; 57°C for 60 s). After amplification, the fluorescence signals of the final PCR products were analyzed using PHERAstar FSX Microplate Reader (BMG Labtech, Germany).

#### PCR-based marker electrophoresis

The 23-bp duplicated sequence located in the exon 2 of the *GBSSI* gene is uniquely representive of glutinous rice (51). The PCR reaction was performed as followed: 95°C for 3 min followed by 35 cycles of 94°C for 30 s, 55°C for 30 s, 72°C for 1 min and final 5 min at 72°C. 196 bp PCR product from Wx-Glu-23bp_F and Wx-Glu-23bp_R primers was detected by 3% agarose gel electrophoresis (Supplementary Table S1).

### Dietary fiber analysis

Dietary fiber was determined by measuring carbohydrates with a degree of polymerization (DP) of more than two, not hydrolysable by endogenous digestive enzymes. The *in vitro* analysis uses heat-stable *α*-amylase, protease, and amyloglucosidase following the AOAC methods 991.43 and 985.29 (K-TDFR, Megazyme) to estimate the dietary fiber content of whole grain rice samples. Briefly, 1 g of finely ground whole-grain rice flour or defatted bran powder was boiled for 30 min with 50 mL of 0.05 M MES/TRIS buffer (pH 8.2) and 0.2 mL of thermostable *α*-amylase (3,000 Units/ml). After cooling, the solution was incubated for 30 min at 60°C with 0.1 mL of protease (50 mg/mL; 350 tyrosine Units/ml) and adjusted to pH 4.5 with 0.561 N HCl. The partially digested flours were further incubated at 60°C for 16 h with 0.2 mL of amyloglucosidase (3,300 Units/ml). The completely digested solution was filtered to separate the insoluble (residue) and soluble (filtrate) fractions. The insoluble residue was washed with 78% ethanol, 95% ethanol and acetone, then dried and weighed to isolate the insoluble dietary fiber (IDF). The weight of the IDF was corrected with crude protein and ash and expressed as a percentage of whole-grain rice flour or rice bran powder. To estimate the SDF, the soluble filtrates were deionized and passed through a column packed with mixed-bed ion exchange resin, concentrated and filtered again through 0.45 μm-membrane filters, and quantified by HPLC with a refractive index detector (Shimadzu RID-10A HPLC system, Shimadzu Corporation, Kyoto, Japan) based on Ohkuma’s method (52) with modification and AOAC methods 2009.01 and 2011.25 (K-INTDF, Megazyme). SDF was expressed as the whole-grain rice flour or bran powder percentage. Total dietary fiber (TDF) was the sum of IDF and SDF.

### Proximate analysis

The iodine-colorimetric method determined the AC using Juliano’s technique (53). Total fat was determined using Soxhlet extraction with petroleum ether, based on AOAC method 945.16. The gravimetric method measured the moisture content found in ISO method 712:2009. Kjeldahl analysis determined crude Protein according to the AOAC method 2001.11. Crude ash was determined by incineration at 525°C for 5 h, according to AOAC method 942.05.

### Alkaline degradation test

The alkaline degradation test, alternatively called Alkaline Spreading Value (ASV), was conducted (54) with minor modifications to estimate the gelatinisation temperature (GT) and alkaline-resistant properties of whole-grain rice samples. Eight whole grain kernels were placed in a closed 10-cm Petri dish containing 20 mL of 1.7% (w/v) KOH aqueous solution for 24 h at room temperature. The alkaline spreading value (ASV) was rated visually on a 7-point numerical scale (1 = intact; 7 = extensively dispersed), and the average scores of eight kernels were taken as the spreading value.

### Textural analysis

Whole grain rice samples were soaked in water – non-waxy rice (1:2 ratio), waxy rice with ASV more than 1 (1:1 ratio), and waxy rice with ASV equal to precisely 1 (1:2 ratio) – in aluminum cups, then cooked in a stainless-steel streamer for 30–45 min until no white starch core was observed before the analysis. A texture profile analysis (TPA) of cooked whole grain rice samples was conducted with a texture analyzer (TA-XT plus, Stable Micro System, Godalming, UK) based on the method used by Parween et al. (20) and Guillen et al. (55), which demonstrated the significant correlation with sensory evaluation by trained panelists. A 50-mm cylinder probe was set at 30 mm above the base. The TPA force-deformation curve was obtained using a two-cycle compression with a force versus distance program. Three warm rice kernels were placed on the base platform under the center of the probe and compressed to 90% of the original cooked grain thickness. Pre-test, test, and post-test speeds were 1, 0.5, and 0.5 mm/s, respectively. Nine measurements were performed for each sample (10 per cup × 3 cups).

### Radar chart imaging

A radar chart was developed to display the complex interrelationship between AC, Dietary fiber Index (DFI), the SDF to IDF ratio, protein, and textural characteristics across rice varieties with high, low, and very low AC (Waxy) using ‘fmsb’ package in R program.

### Statistical analysis

The analysis was performed using Statgraphics Centurion XVII software (Statpoint Technologies, Warrenton, VA, USA). The data were analyzed in triplicate by one-way analysis of variance, and Duncan’s multiple range test was used to determine statistically significant differences among the samples. These differences were indicated by different letters in the columns when the *p*-value was lower than 0.05. Linear regression was also analyzed using the Pearson correlation two-tailed test, with a significance level of 0.05 and 0.01. A correlation matrix was developed to identify the correlations between two variables, using a linear relationship Pearson correlation coefficient at a significance level of 0.05 and 0.01.

We performed Structural Equation Modeling (SEM), an integrated factor and multiple regression analyses, to quantify the structural relationships between causes and effects. All selected variables were standardized to have a mean of 0 and a standard deviation of 1 before the analysis. SEM involved the R Software Platform (56) and the Lavaan Package (57). Path coefficients were partitioned into direct and indirect effect components using Wrigh’s method (58) to illustrate the relationship between correlation and path coefficients. Any missing data were corrected using a pairwise deletion. After creating the SEM, the Adjusted Goodness-of-Fit Statistic (AGFI >0.90) and the Standardized Root Mean Square Residual (SRMR <0.05) were utilized to fit the overall model (59). Only SEM with R-square values below 60 were dropped for further interpretation. All scripts for the analyses were listed in the Supplementary Table S2.

## Results

### Genetic and nutritional diversity

**Figure 1:**
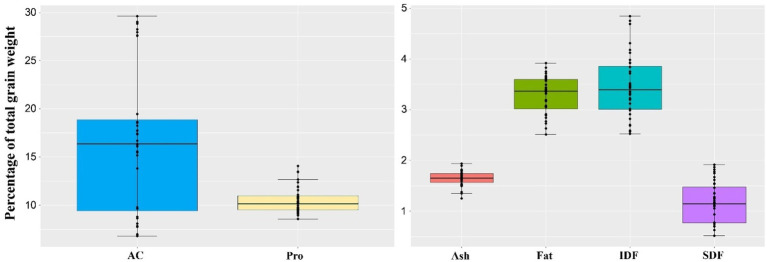
Overall variation in nutritional components of whole-grain pigmented rice samples varying in AC, expressed as a percentage of total grain weight; AC: % Amylose, PRO: % Protein, FAT: % Fat, ASH: % Ash, SDF: % soluble dietary fiber, IDF: % insoluble dietary fiber, CHO: Available carbohydrate from the calculation [100 – (Protein + fat + ash + total dietary fiber)].

The purple rice population contained significant variations in AC and ASV, followed by IDF and SDF. AC ranged from 6.81 to 29.6% and alkaline spreading value (ASV) from 1.0 to 5.4 (Figure 1 and Supplementary Table S3). We classified rice varieties based on AC and allele-specific SNP markers into waxy (AC < 12%), low amylose (AC from 12 to 20%), and high amylose (AC > 20%). For AC, the G/T SNP and 23-bp insertion precisely classified the 36 rice varieties into high (8), low (17), and waxy (11) groups. Within each AC group, rice varieties varied considerably within the specified ranges of AC (Supplementary Table S3). In this study, the SNP markers showed high accuracy even at the borderline between high vs. low AC and low AC vs. waxy groups (Supplementary Tables S3, S5). In contrast, the GC/TT SNP marker and ASV showed less association with the whole-grain purple rice in this study.

**Figure 2:**
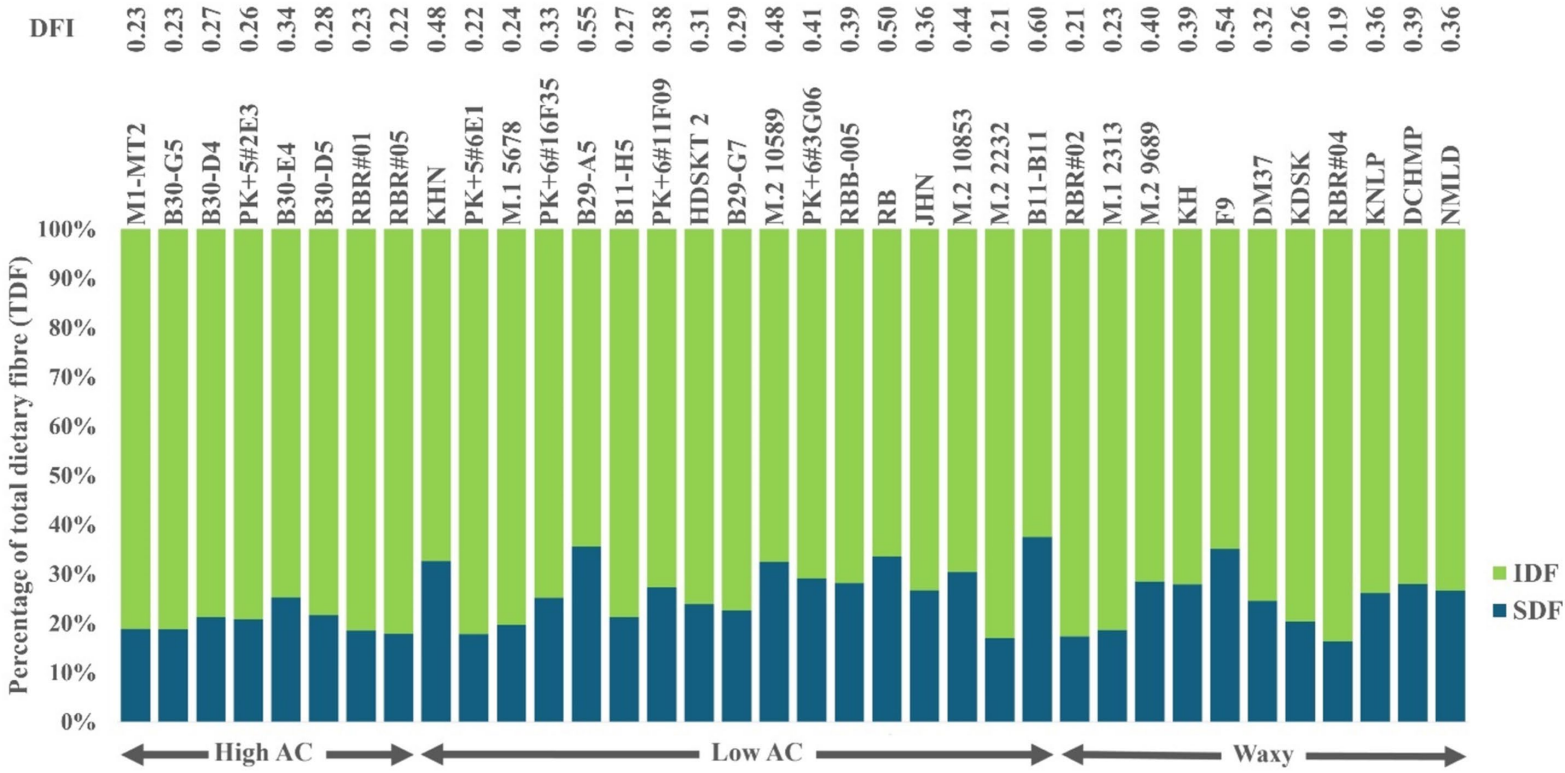
Variation in Dietary Fiber Indexes (DFI), the ratio of SDF to IDF, expressed as a percentage of TDF, among whole-grain pigmented rice varieties with different AC.

The DFI, the SDF/IDF ratio, ranged from 0.19 to 0.60 (Figure 2). In particular, high DFI rice varieties were primarily found among low AC rice varieties. In comparison, high DFI rice >50 mainly contained a lower IDF or higher SDF than lower DFI rice. In contrast, high IDF varieties were found only in the waxy rice group (Supplementary Table S3). In addition, the average protein content was distributed narrowly but in a higher range, while fat content was equally distributed among the whole-grain purple rice varieties. Interestingly, purple rice’s protein contents were exceptionally higher among waxy and low AC groups, ranging from 8.56 to 14.09% (Supplementary Table S3). Such a collection of purple rice represents the extreme genetic diversity suitable for studying the impacts of nutrients on cooked rice quality.

**Figure 3:**
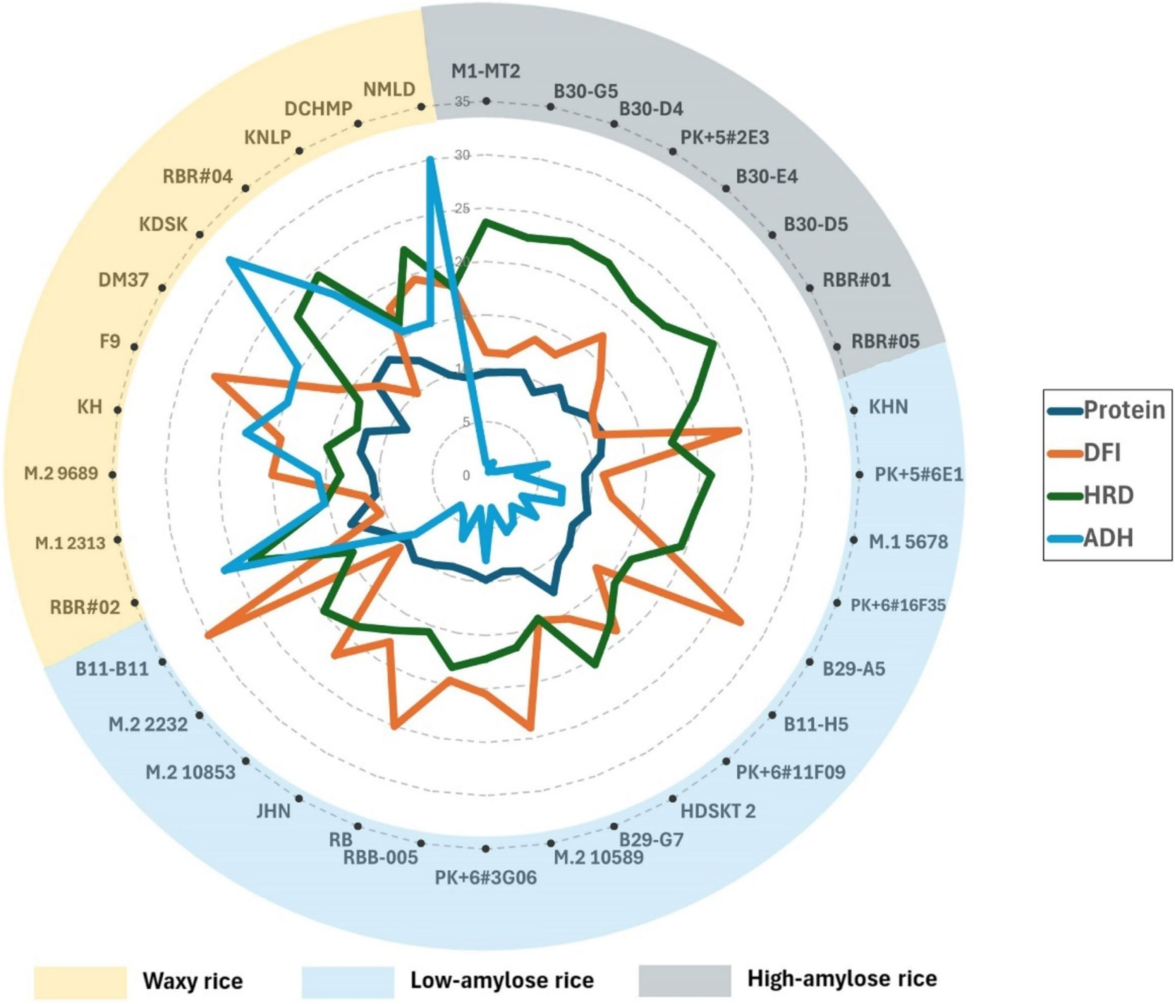
Radar chart showing relationships between hardness (HRD) and adhesiveness (ADH) of cooked whole-grain rice and AC and AP (DFI), protein of whole-grain pigmented rice with high amylose (AC > 20%), Low Amylose (AC > 12–20%), and Waxy (AC < 12%). Rice varieties are sorted clockwise from high to low AC.

Within each AC group, rice varieties varied in nutrient parameters, particularly DFI and protein content, which could affect the textural characteristics of whole-grain rice, particularly HRD and ADH. AC strongly affected HRD among the high AC group, which shows a narrow variation in DFI. DFI had little effect on HRD but was significantly high in ADH (Figure 3 and Supplementary Tables S3, S4). The B30-E4, with the highest DFI at 0.34 among the high AC group, showed a slightly lower HRD but significantly lower ADH than the group (Figure 3 and Supplementary Tables S3, S4). Besides regular green-leaf rice varieties, we showed that two highly pigmented-leaf Rainbow Rice varieties, RBR#01 and RBR#05, had high AC and HRD with low DFI at 0.22–0.23.

In contrast, DFI showed a considerable variation among the low AC groups and exhibited a good relationship with HRD—all four high DFI rice varieties, *viz.* RB, B11-11, KHN, and M.210589 showed high DFI with similar low HRD (Figure 3 and Supplementary Tables S3, S4). In contrast, waxy purple rice varieties generally showed lower DFI but higher HRD than the low AC group, with a few exceptions (Figure 3). F9 was exceptionally low in IDF, resulting in high DFI (Supplementary Tables S3, S4) but low HRD (Figure 3). Nonetheless, the two waxy Rainbow Rice varieties, RBR#02 and RBR#04, with the two top protein content at 13.43 and 14%, respectively, were among the lowest DFI at 0.21 and 0.19, respectively, but high contrast in ADH but similar HRD (Figure 3). Besides the influence of AC, DFI and protein also play roles in HRD and ADH.

For the average 10.5% whole-grain protein content, the purple rice germplasm showed superior seed protein than commercial varieties of brown-colored rice. Notably, waxy purple rice contained higher protein than those low and high-AC purple rice groups (Supplementary Tables S3, S4). RBR#02 and RBR#04, Rainbow Rice varieties, were among the top protein contenders among the waxy rice group (Figure 3 and Supplementary Tables S3, S4). In addition, KDSK, KH, and F9 were among the waxy rice varieties with >12% protein content. Nonetheless, only two high-protein rice varieties reported in non-waxy rice groups were HDSKT 2 (>12% protein, low AC) and RBR#05 (11.48% protein, high AC). Understanding the impacts of protein on HRD and ADH is interesting in this purple rice germplasm.

Protein corresponded well with HRD and ADH among waxy and low-AC rice varieties. In contrast, AC strongly affected HRD and ADH more than protein content in the high AC group but not in the low and waxy groups (Figure 3). Variation in ADH was also significant among waxy rice but less among low AC rice varieties. Nonetheless, ADH varied considerably in the low AC rice varieties, albeit less than in the waxy group, but showed an inconsistent relationship with protein contents. For instance, HDSKT 2, the top protein content among the low AC group, was the least ADH. While RB and B11-B11 differed by less than 1% protein content among the low AC group, they showed a substantial contrast in ADH. RBR#05 (high AC) and PK + 6#3G06 (low AC) differed by less than 1% in protein content but were significantly high in ADH. Still, the difference in AC affected ADH to some degree. We found a more consistent relationship between protein content, DFI and ADH among waxy rice varieties. Interestingly, waxy rice F9, with 11.9% protein content, showed the highest DFI and softest cooked rice among whole-grain purple rice varieties (Figure 3 and Supplementary Tables S3, S4). Therefore, we were interested in dissecting the relationship between protein content, dietary fiber, starch and textural characteristics of whole-grain purple rice through binary correlation and multiple regression analyses.

### Pearson correlation analysis

**Figure 4:**
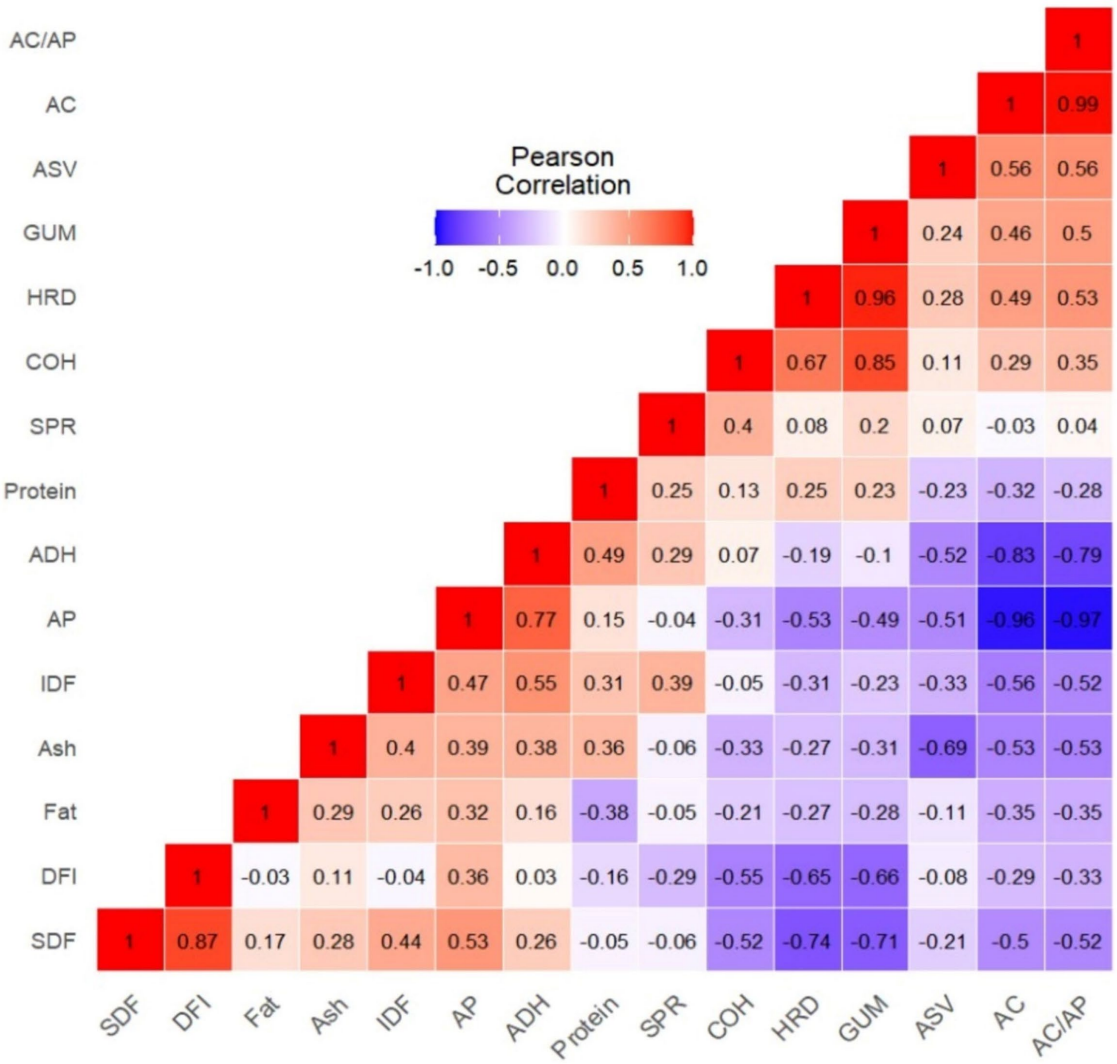
Pearson’s correlation coefficient matrix among amylose content, nutritional components, dietary fiber profiles, and textural parameters. AC, amylose content; AP, Amylopectin (Starch – AC); SDF, soluble dietary fiber; IDF, insoluble dietary fiber; DFI, SDF to IDF ratio; HRD, hardness; ADH, adhesiveness; SPR, springiness; COH, cohesiveness; GUM, gumminess; CHEW, chewiness.

Correlation analysis demonstrated how complex nutritional components, including starch, dietary fiber, and protein, pairwise interacted with the textural characteristics of whole-grain purple rice varieties (Figure 4). AC, AP, and AC/AP strongly influenced HRD, ADH, and GUM but weakly with COH, whereas SDF, IDF, and DFI were strongly associated with the overall textural characteristics. Specifically, SDF and DFI had a significantly negative association with HRD, COH, GUM, and Chew, while IDF was associated strongly with ADH but weakly with SPR. SDF and DFI showed a significant association, but IDF and DFI were independent. Therefore, variation in SDF, not IDF, affected DFI. Conversely, protein content significantly correlated with ADH but not other nutrient factors and textural attributes (Figure 4). We may conclude from correlation analysis that protein did not affect textural characteristics in the purple rice germplasm.

### Path coefficient analysis

A pairwise correlation analysis, like the Pearson correlation coefficients, only determines the binary relationship between two variables, regardless of differentiating which independent variables have direct or indirect effects on a dependent variable or how strongly independent variables affect a dependent variable. We designed a Path Coefficient Analysis (PCA) to determine the directions and impacts of nutrient factors (independent variables) on the textural characteristics (dependent variables) in cooked whole-grain purple rice.

**Figure 5:**
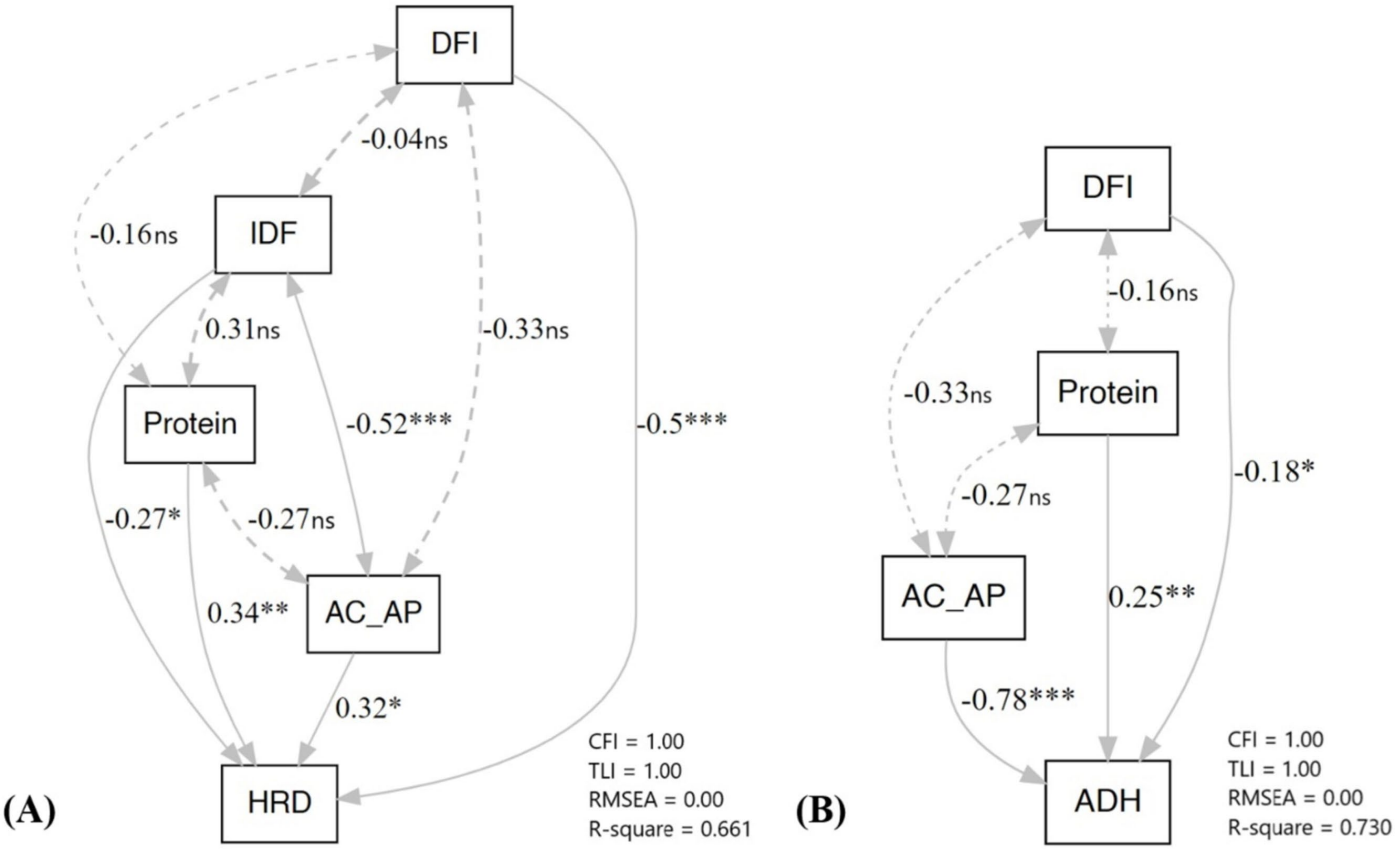
Path Coefficient Analysis (PCA) determines **(A)** Hardness (HRD) and **(B)** Adhesiveness (ADH).

Cooked Rice Hardness (HRD): The Pearson correlation indicated that the AC and AC/AP variation showed positive influences, but AP, SDF, and DFI were negatively associated with HRD. Nonetheless, PCA revealed that DFI (−0.5, *p* < 0.01) had the most potent negative direct effect, followed by IDF (−0.27, *p* > 0.05), whereas protein (0.34, *p* < 0.05) and AC/AP (0.32, *p* > 0.05) directly and positively affected HRD (Figure 5A). The fact that Pearson correlation analysis failed to detect the association between Protein and HRD, PCA strengthened the correlation analysis with the power of differentiation between the actual cause and effect. Although IDF contributed a weaker direct effect on HRD, it exerted a more substantial negative indirect effect via AC/AP and could balance protein effects on HRD. Our results inferred that low AC or waxy pigmented rice containing higher protein and DFI tend to be softer than rice with lower DFI.

Adhesiveness (ADH): From the correlation coefficient, ADH was associated with AP, IDF, and protein but negatively associated with AC, AC/AP, and ASV. However, the PCA revealed AC/AP strongly negatively impacted ADH, followed by DFI, while protein positively affected ADH (Figure 5B).

COH, GUM, SPR, and CHEW: Because their R-square values of the SEM were too low, we did not consider them significant for further investigation (Supplementary Tables S10–S17).

## Discussion

In the previous study, 25 non-pigmented and pigmented rice varieties revealed the critical roles of DFI and AC on the textural characteristics of cooked whole-grain rice using a pairwise correlation analysis (47). This study investigated 36 whole-grain purple rice varieties, comprising wider variations in DFI, AC, protein and textural attributes, using the Pearson correlation coefficient and Path-coefficient analysis (PCA). Because Pearson’s correlation coefficient, a bivariate analysis, cannot explain the actual causal effects of seed nutrient composition on textural attributes, we introduced Path-coefficient Analysis to dissect the nutrient components, the independent variables, into direct and indirect factors on the textural characteristics, the dependent variable, using Structural Equation Modeling (SEM), a multivariate statistical technique. We successfully quantified the structural relationships between causes and effects in a single study based on factor analysis and multiple regression (60). Unlike the Pearson correlation coefficient, SEM identifies which variables directly or indirectly impact the attribute.

Using Path Coefficient Analysis (PCA), we dissected the complex interaction between DFI, protein and AC/AP in HRD. The results clearly show that protein and AC/AP positively impacted HRD. Still, dietary fiber, including DFI and IDF, directly opposed the impact of protein and AC/AP on HRD in whole-grain purple rice. The outcome demonstrated for the first time the crucial roles of soluble and insoluble dietary fibers in antagonizing the influence of protein on HRD of whole-grain rice among low and waxy rice varieties. The outcome opens a window of opportunity to enhance protein content without affecting the textural quality of whole-grain rice among low and waxy rice varieties.

In contrast, only protein positively impacted ADH, while AC/AP and DFI opposed the impact of protein on the adhesiveness of cooked whole-grain purple rice. Several studies have also reported the effect of protein on HRD of polished white rice varieties which contain no dietary fiber (21–23). Higher endosperm protein content increased the HRD and reduced ADH (61). Protein and short-chain AC were strongly positively correlated with HRD but had less effect on ADH (21, 22). During cooking, a protein-starch network disturbs water absorption and pasting quality, particularly during steaming, due to reduced starch granule swelling (21, 23), which influences cooked rice hardness (18). Consequently, white rice with high-protein content is harder, less sticky, and less elastic than lower-protein white rice (24, 25, 62).

Whole-grain rice contains 50% more total dietary fiber (TDF) than white rice, suggesting that most dietary fiber is in the bran fraction (43, 63, 64). Most TDF in whole-grain rice is insoluble dietary fiber (IDF), ranging from 2.53–4.84%, while soluble fiber (SDF) constitutes only 1.17% on average, with a slight variation (47). Growing evidence indicates that consuming whole-grain rice prevents the risk of type-2-diabetes (T2D) (9, 12, 65). Despite the health benefits, high dietary fiber affected the softness of cooked whole-grain rice and consumer acceptance (15, 43–45). In contrast, as reflected by high DFI, whole-grain rice with high SDF is softer than low-DFI rice (47). Understanding the interaction between dietary fiber, protein, and starch is crucial for developing palatable whole-grain rice, supporting future global nutrient security, and guarding against NCDs.

## Conclusion

This study investigated the effects of nutritional components, especially protein, dietary fiber and amylose/amylopectin, on the textural properties of cooked whole-grain pigmented rice using Path Coefficient Analysis. Results demonstrated that seed protein and AC/AP positively impacted cooked whole-grain hardness. In contrast, the DFI, soluble to insoluble dietary fiber (SDF/IDF) ratio, offset the impacts of protein and AC/AP on hardness. Concurrently, AC/AP negatively impacted ADH, followed by DFI, while protein positively affected ADH of cooked whole-grain pigmented rice. This finding is helpful for future trends to improve softness and stickiness for better consumer acceptance of high protein whole-grain pigmented rice. With their enrichment in phenolic compounds, protein and dietary fiber, consuming whole-grain pigmented rice could help slow down the incidence of metabolic syndrome among half of the global population consuming rice daily.

## Data Availability

The datasets presented in this study can be found in online repositories. The names of the repository/repositories and accession number(s) can be found in the article/Supplementary material.

## Footnotes

^1^ http://www.lgcgenomics.com
