# Supplementary material for: Path coefficient analysis unraveled nutrient factors directly impacted the textural characteristics of cooked whole-grain purple rice
Source: Front Nutr. 2024 Oct 29;11:1490404. doi: 10.3389/fnut.2024.1490404 (PMC11554458; doi:10.3389/fnut.2024.1490404)
Supplement: Supplementary file 1 [file Table_1.docx]

Supplementary Material

**Supplementary Table S1.** KASP SNP markers for specific gene target and PCR based marker electrophoresis used for genotyping.

| **Traits** | **Marker Name** | **Chr.** | **QTL/gene** | **LGC code** | **Reference** |
| --- | --- | --- | --- | --- | --- |
| Amylose content (AC) | wx_5UTR_G/T | 6 | GBSSI | 002–0052.1 | Vanavichit et al., 2018 |
| Gelatinisation temperature (GT) | ALK_ex8_SNP_GC/TT | 6 | SSIIa | 002–0049.1 | Vanavichit et al., 2018 |
| 23 bp duplicated (glutinous) | Wx-Glu-23bp | 6 | GBSSI | - | Wanchana et al., 2003 |

**Supplementary Table S2.** Listed of all scripts for the analyses using R program.

| **#install_packages** | **#Modeling** | *****GUM** |
| --- | --- | --- |
| installed.packages("lavaan") | *****HRD** | model<-' |
| install.packages("lavaan") | model<-' | GUM ~ AC + Protein + DFI |
| install.packages("htmltools") | HRD ~ AC_AP + Protein + IDF + DFI | AC ~~ Protein + DFI |
| install.packages("semPlot") | AC_AP ~~ Protein + IDF + DFI | Protein ~~ DFI |
| install.packages("lavaanPlot") | Protein ~~ IDF + DFI | ' |
| install.packages("sjPlot") | IDF ~~ DFI |  |
| install.packages("Hmisc") | ' | *****CHEW** |
| install.packages("agricolae") |  | model<-' |
| library("lavaan") | *****ADH** | CHEW ~ AC + AC_AP + DFI |
| library("semPlot") | model<-' | AC ~~ AC_AP + DFI |
| library("lavaanPlot") | ADH ~ AC_AP + Protein + DFI | AC_AP ~~ DFI |
| library("readxl") | AC_AP ~~ Protein + DFI | ' |
| library("sjPlot") | Protein ~~ DFI |  |
| library("Hmisc") | ' | **#Pearson_all** |
| library("agricolae") |  | tab_corr(raw_data[,c(2,3,4,6,7,8,9,10,11,12,13,14,15,16,17,18)]) |
|  | *****SPR** |  |
| **#import data** | model<-' |  |
| raw_data <- read_excel("PCA/Raw_data_new1.xlsx") | SPR ~ AC + AC_AP + IDF |  |
| View(raw_data) | AC ~~ AC_AP + IDF |  |
|  | AC_AP ~~ IDF |  |
| **#direct and indirect effect** | ' |  |
| x<-raw_data[,c(4,7,10,11)] |  |  |
| y<-raw_data[,13] | *****COH** | **#Estimated and summarised** |
| data <- cbind(y,x) | model<-' | fit <- sem(model, data = raw_data) |
| cor.x <- rcorr(as.matrix(x))$r | COH ~ Ash + DFI | summary(fit,standardized=T,fit=T,rsquare=T) |
| cor.y <- as.data.frame(t(subset(rcorr(as.matrix(cbind(y,x)))$r, select = c(HRD))))[,-1] | Ash ~~ DFI | sem.model <- lavaanPlot(model = fit, node_options = list(shape = "box", fontname = "Helvetica"), edge_options = list(color = "grey"), coefs = TRUE, stand = TRUE,sig=0.05,stars = c("regress", "covs"),covs=T) |
| Path <- path.analysis(cor.x,cor.y) | ' | print(sem.model) |
| diag(Path$Coeff) |  |  |
| Path$Residual |  |  |

**Supplementary Table S3.** Proximate analysis values with standard deviation (+SD) as a whole grain rice flour percentage from selected pigmented rice varieties.

| **Varieties** | **Amylose** | | | **Starch** | | | **Fat** | | | **Protein** | | | **Ash** | | | **SDF** | | | **IDF** | | | **DFI** | **ASV** |
| --- | --- | --- | --- | --- | --- | --- | --- | --- | --- | --- | --- | --- | --- | --- | --- | --- | --- | --- | --- | --- | --- | --- | --- |
| M1-MT2 | 29.60 | ± | 0.09 | 79.29 | ± | 0.32 | 2.51 | ± | 0.04 | 9.59 | ± | 0.02 | 1.51 | ± | 0.01 | 0.78 | ± | 0.13 | 3.35 | ± | 0.06 | 0.23 | 1.0 |
| B30-G5 | 29.01 | ± | 0.02 | 78.59 | ± | 0.23 | 3.18 | ± | 0.01 | 9.79 | ± | 0.09 | 1.25 | ± | 0.00 | 0.70 | ± | 0.02 | 3.02 | ± | 0.16 | 0.23 | 5.4 |
| B30-D4 | 29.00 | ± | 0.05 | 78.75 | ± | 0.16 | 2.90 | ± | 0.02 | 10.24 | ± | 0.05 | 1.35 | ± | 0.00 | 0.70 | ± | 0.01 | 2.59 | ± | 0.00 | 0.27 | 3.6 |
| PK+5#2E3 | 28.84 | ± | 0.46 | 77.83 | ± | 0.16 | 3.39 | ± | 0.05 | 8.93 | ± | 0.03 | 1.67 | ± | 0.01 | 0.71 | ± | 0.02 | 2.69 | ± | 0.03 | 0.26 | 1.0 |
| B30-E4 | 28.25 | ± | 0.02 | 75.91 | ± | 0.02 | 3.19 | ± | 0.00 | 10.72 | ± | 0.16 | 1.50 | ± | 0.00 | 1.18 | ± | 0.03 | 3.49 | ± | 0.01 | 0.34 | 4.0 |
| B30-D5 | 27.96 | ± | 0.04 | 77.08 | ± | 0.43 | 3.44 | ± | 0.10 | 9.75 | ± | 0.00 | 1.38 | ± | 0.00 | 0.71 | ± | 0.01 | 2.57 | ± | 0.08 | 0.28 | 4.0 |
| RBR#01 | 27.64 | ± | 0.20 | 77.55 | ± | 0.14 | 2.89 | ± | 0.07 | 11.08 | ± | 0.03 | 1.68 | ± | 0.00 | 0.78 | ± | 0.11 | 3.43 | ± | 0.08 | 0.23 | 2.3 |
| RBR#05 | 27.58 | ± | 0.17 | 75.76 | ± | 0.11 | 2.84 | ± | 0.04 | 11.58 | ± | 0.02 | 1.71 | ± | 0.01 | 0.75 | ± | 0.01 | 3.45 | ± | 0.05 | 0.22 | 1.7 |
| KHN | 19.46 | ± | 0.02 | 74.77 | ± | 0.19 | 3.41 | ± | 0.05 | 10.92 | ± | 0.14 | 1.79 | ± | 0.01 | 1.55 | ± | 0.08 | 3.20 | ± | 0.10 | 0.48 | 1.9 |
| PK+5#6E1 | 18.67 | ± | 0.03 | 77.42 | ± | 0.19 | 3.72 | ± | 0.01 | 9.27 | ± | 0.02 | 1.63 | ± | 0.07 | 0.70 | ± | 0.02 | 3.23 | ± | 0.00 | 0.22 | 1.5 |
| M.1 5678 | 18.55 | ± | 0.08 | 75.47 | ± | 0.01 | 3.59 | ± | 0.05 | 9.48 | ± | 0.01 | 1.73 | ± | 0.00 | 0.63 | ± | 0.04 | 2.57 | ± | 0.13 | 0.24 | 1.7 |
| PK+6#16F35 | 18.23 | ± | 0.12 | 76.20 | ± | 0.11 | 3.57 | ± | 0.11 | 10.15 | ± | 0.01 | 1.71 | ± | 0.05 | 1.11 | ± | 0.05 | 3.30 | ± | 0.03 | 0.33 | 1.2 |
| B29-A5 | 17.78 | ± | 0.01 | 79.78 | ± | 0.29 | 2.91 | ± | 0.18 | 9.38 | ± | 0.04 | 1.54 | ± | 0.01 | 1.78 | ± | 0.00 | 3.22 | ± | 0.00 | 0.55 | 2.6 |
| B11-H5 | 17.73 | ± | 0.05 | 79.02 | ± | 0.32 | 3.06 | ± | 0.03 | 10.18 | ± | 0.03 | 1.62 | ± | 0.02 | 1.06 | ± | 0.49 | 3.92 | ± | 0.22 | 0.27 | 2.4 |
| PK+6#11F09 | 17.42 | ± | 0.17 | 74.25 | ± | 0.07 | 2.77 | ± | 0.07 | 10.65 | ± | 0.11 | 1.51 | ± | 0.04 | 1.25 | ± | 0.10 | 3.32 | ± | 0.05 | 0.38 | 2.4 |
| HDSKT 2 | 17.37 | ± | 0.03 | 75.51 | ± | 0.11 | 3.05 | ± | 0.02 | 12.67 | ± | 0.04 | 1.74 | ± | 0.02 | 0.94 | ± | 0.12 | 2.99 | ± | 0.14 | 0.31 | 1.3 |
| B29-G7 | 16.68 | ± | 0.03 | 76.65 | ± | 0.50 | 3.32 | ± | 0.01 | 9.54 | ± | 0.05 | 1.58 | ± | 0.02 | 1.10 | ± | 0.06 | 3.75 | ± | 0.05 | 0.29 | 2.2 |
| M.2 10589 | 16.38 | ± | 0.08 | 77.49 | ± | 0.19 | 3.64 | ± | 0.04 | 9.15 | ± | 0.02 | 1.65 | ± | 0.01 | 1.92 | ± | 0.10 | 3.98 | ± | 0.63 | 0.48 | 2.2 |
| PK+6#3G06 | 16.31 | ± | 0.13 | 76.44 | ± | 0.36 | 3.17 | ± | 0.14 | 9.80 | ± | 0.11 | 1.53 | ± | 0.03 | 1.16 | ± | 0.00 | 2.82 | ± | 0.03 | 0.41 | 1.3 |
| RBB-005 | 16.14 | ± | 0.14 | 76.19 | ± | 0.13 | 3.67 | ± | 0.03 | 9.19 | ± | 0.01 | 1.63 | ± | 0.02 | 1.54 | ± | 0.14 | 3.93 | ± | 0.15 | 0.39 | 2.3 |
| RB | 16.12 | ± | 0.13 | 75.57 | ± | 0.31 | 3.41 | ± | 0.05 | 9.10 | ± | 0.10 | 1.81 | ± | 0.15 | 1.75 | ± | 0.03 | 3.46 | ± | 0.07 | 0.50 | 2.5 |
| JHN | 15.59 | ± | 0.24 | 78.18 | ± | 0.21 | 3.63 | ± | 0.01 | 9.70 | ± | 0.09 | 1.65 | ± | 0.00 | 1.14 | ± | 0.00 | 3.12 | ± | 0.11 | 0.36 | 2.8 |
| M.2 10853 | 15.48 | ± | 0.09 | 76.36 | ± | 0.22 | 3.37 | ± | 0.06 | 10.34 | ± | 0.06 | 1.76 | ± | 0.00 | 1.27 | ± | 0.01 | 2.91 | ± | 0.37 | 0.44 | 2.3 |
| M.2 2232 | 15.17 | ± | 0.13 | 74.65 | ± | 0.29 | 3.65 | ± | 0.07 | 9.55 | ± | 0.03 | 1.82 | ± | 0.03 | 0.52 | ± | 0.07 | 2.53 | ± | 0.06 | 0.21 | 1.4 |
| B11-B11 | 13.81 | ± | 0.09 | 78.22 | ± | 0.15 | 2.63 | ± | 0.02 | 10.83 | ± | 0.02 | 1.74 | ± | 0.01 | 1.62 | ± | 0.10 | 2.70 | ± | 0.03 | 0.60 | 1.0 |
| RB 02 | 9.80 | ± | 0.17 | 74.39 | ± | 0.02 | 2.73 | ± | 0.02 | 13.43 | ± | 0.01 | 1.64 | ± | 0.01 | 0.78 | ± | 0.01 | 3.72 | ± | 0.00 | 0.21 | 2.0 |
| M.1 2313 | 9.65 | ± | 0.02 | 74.98 | ± | 0.15 | 3.49 | ± | 0.03 | 10.47 | ± | 0.04 | 1.81 | ± | 0.00 | 1.11 | ± | 0.02 | 4.84 | ± | 0.12 | 0.23 | 1.0 |
| M.2 9689 | 8.80 | ± | 0.05 | 73.15 | ± | 0.20 | 3.92 | ± | 0.20 | 10.65 | ± | 0.03 | 1.74 | ± | 0.03 | 1.87 | ± | 0.05 | 4.69 | ± | 0.13 | 0.40 | 1.1 |
| KH | 8.66 | ± | 0.08 | 71.42 | ± | 0.49 | 3.37 | ± | 0.02 | 11.92 | ± | 0.01 | 1.81 | ± | 0.01 | 1.67 | ± | 0.00 | 4.31 | ± | 0.25 | 0.39 | 1.0 |
| F9 | 8.10 | ± | 0.03 | 76.54 | ± | 0.07 | 2.77 | ± | 0.01 | 11.90 | ± | 0.23 | 1.61 | ± | 0.13 | 1.82 | ± | 0.02 | 3.36 | ± | 0.05 | 0.54 | 1.0 |
| DM37 | 7.85 | ± | 0.10 | 76.22 | ± | 0.29 | 3.83 | ± | 0.01 | 8.56 | ± | 0.05 | 1.66 | ± | 0.00 | 1.36 | ± | 0.00 | 4.18 | ± | 0.09 | 0.32 | 1.0 |
| KDSK | 7.85 | ± | 0.10 | 72.66 | ± | 0.18 | 3.35 | ± | 0.02 | 13.49 | ± | 0.01 | 1.90 | ± | 0.00 | 1.22 | ± | 0.00 | 4.75 | ± | 0.20 | 0.26 | 1.0 |
| RBR#04 | 7.76 | ± | 0.02 | 71.04 | ± | 0.22 | 3.08 | ± | 0.01 | 14.09 | ± | 0.01 | 1.69 | ± | 0.02 | 0.75 | ± | 0.12 | 3.84 | ± | 0.16 | 0.20 | 1.4 |
| KNLP | 6.98 | ± | 0.06 | 72.81 | ± | 0.49 | 3.61 | ± | 0.08 | 12.38 | ± | 0.00 | 1.94 | ± | 0.00 | 1.46 | ± | 0.01 | 4.12 | ± | 0.07 | 0.36 | 1.1 |
| DCHMP | 6.95 | ± | 0.02 | 78.24 | ± | 0.15 | 3.44 | ± | 0.15 | 9.98 | ± | 0.03 | 1.61 | ± | 0.01 | 1.35 | ± | 0.05 | 3.47 | ± | 0.06 | 0.39 | 2.0 |
| NMLD | 6.81 | ± | 0.01 | 78.80 | ± | 0.06 | 3.75 | ± | 0.19 | 9.25 | ± | 0.18 | 1.50 | ± | 0.01 | 1.28 | ± | 0.42 | 3.52 | ± | 0.48 | 0.36 | 1.4 |

Results are the means ± SE on dry basis and expressed as the whole grain rice flour percentage. Values with different letters in the same column are significantly different with P < 0.05. Abbreviations: SDF = soluble dietary fibre; IDF = insoluble dietary fibre; DFI = soluble/insoluble dietary fibre; ASV = alkaline spreading value

**Supplementary Table S4.** Texture profile values with standard deviation (+SD) of cooked whole grain pigmented rice samples.

| Varieties | Hardness (N) | Adhesiveness (mN.sec) | Springiness (Sec/Sec) | Cohesiveness (N.sec/N.sec) | Gumminess (N) | Chewiness (N) |
| --- | --- | --- | --- | --- | --- | --- |
| M1-MT2 | 23.66 ± 0.21 | 5.79 ± 0.84 | 0.80 ± 0.01 | 0.52 ± 0.00 | 12.31 ± 0.17 | 9.92 ± 0.14 |
| B30-G5 | 22.55 ± 0.52 | 5.64 ± 0.87 | 0.82 ± 0.02 | 0.46 ± 0.01 | 10.37 ± 0.45 | 8.40 ± 0.21 |
| B30-D4 | 23.23 ± 0.93 | 3.76 ± 0.55 | 0.80 ± 0.05 | 0.47 ± 0.01 | 10.99 ± 0.60 | 8.70 ± 0.42 |
| PK+5#2E3 | 22.94 ± 1.12 | 6.84 ± 0.54 | 0.86 ± 0.06 | 0.51 ± 0.02 | 11.85 ± 0.86 | 10.352 ± 1.10 |
| B30-E4 | 21.49 ± 0.43 | 2.02 ± 1.03 | 1.04 ± 0.15 | 0.45 ± 0.00 | 9.69 ± 0.27 | 9.74 ± 1.02 |
| B30-D5 | 21.76 ± 1.74 | 4.04 ± 0.67 | 0.87 ± 0.02 | 0.48 ± 0.03 | 10.55 ± 1.51 | 9.03 ± 1.19 |
| RBR#01 | 24.57 ± 0.99 | 3.49 ± 0.26 | 0.78 ± 0.03 | 0.45 ± 0.01 | 11.15 ± 0.60 | 8.69 ± 0.76 |
| RBR#05 | 20.84 ± 0.46 | 4.98 ± 0.20 | 0.82 ± 0.00 | 0.42 ± 0.01 | 8.86 ± 0.23 | 7.18 ± 0.14 |
| KHN | 17.66 ± 0.23 | 29.18 ± 4.01 | 0.69 ± 0.01 | 0.39 ± 0.01 | 6.99 ± 0.20 | 4.82 ± 0.04 |
| PK+5#6E1 | 21.11 ± 0.88 | 14.69 ± 1.94 | 0.71 ± 0.02 | 0.46 ± 0.01 | 9.90 ± 0.61 | 7.11 ± 0.59 |
| M.1 5678 | 19.85 ± 0.12 | 36.17 ± 2.06 | 0.69 ± 0.01 | 0.44 ± 0.00 | 8.87 ± 0.02 | 6.15 ± 0.13 |
| PK+6#16F35 | 19.45 ± 0.26 | 37.62 ± 6.20 | 0.72 ± 0.03 | 0.42 ± 0.01 | 8.29 ± 0.08 | 6.02 ± 0.19 |
| B29-A5 | 15.76 ± 0.65 | 19.49 ± 1.91 | 0.74 ± 0.03 | 0.43 ± 0.01 | 6.74 ± 0.42 | 5.14 ± 0.42 |
| B11-H5 | 15.76 ± 0.76 | 30.46 ± 6.89 | 1.06 ± 0.45 | 0.41 ± 0.01 | 6.57 ± 0.49 | 8.46 ± 4.68 |
| PK+6#11F09 | 18.11 ± 0.31 | 18.92 ± 4.49 | 0.68 ± 0.01 | 0.41 ± 0.00 | 7.38 ± 0.20 | 4.99 ± 0.20 |
| HDSKT 2 | 20.46 ± 0.39 | 25.76 ± 1.14 | 0.68 ± 0.02 | 0.39 ± 0.01 | 8.02 ± 0.27 | 5.41 ± 0.27 |
| B29-G7 | 14.26 ± 0.71 | 28.38 ± 2.07 | 0.73 ± 0.01 | 0.39 ± 0.01 | 5.69 ± 0.44 | 4.21 ± 0.32 |
| M.2 10589 | 16.39 ± 0.31 | 15.38 ± 1.13 | 0.59 ± 0.06 | 0.39 ± 0.01 | 6.41 ± 0.30 | 3.84 ± 0.58 |
| PK+6#3G06 | 17.20 ± 0.78 | 39.82 ± 0.86 | 0.64 ± 0.03 | 0.39 ± 0.01 | 6.11 ± 0.23 | 3.92 ± 0.34 |
| RBB-005 | 18.25 ± 0.41 | 15.82 ± 2.27 | 0.73 ± 9.92 | 0.42 ± 0.00 | 7.81 0.23 | 5.83 ± 0.32 |
| RB | 15.6 ± 0.13 | 31.73 ± 10.91 | 0.7 ± 0.04 | 0.39 ± 0.01 | 6.21 ± 0.13 | 4.39 ± 0.18 |
| JHN | 16.78 ± 0.84 | 20.85 ± 3.32 | 0.63 ± 0.05 | 0.40 ± 0.01 | 6.73 ± 0.52 | 4.38 ± 0.62 |
| M.2 10853 | 18.51 ± 0.29 | 18.33 ± 1.40 | 0.70 ± 0.02 | 0.40 ± 0.01 | 7.36 ± 0.20 | 5.16 ± 0.25 |
| M.2 2232 | 19.76 ± 0.64 | 43.02 ± 5.10 | 0.69 ± 0.02 | 0.41 ± 0.01 | 8.11 ± 0.49 | 5.72 ± 0.31 |
| B11-B11 | 14.37 ± 0.35 | 61.97 ± 5.12 | 0.63 ± 0.03 | 0.41 ± 0.00 | 5.97 ± 0.14 | 3.87 ± 0.26 |
| RB 02 | 23.33 ± 0.62 | 130.16 ± 18.61 | 0.88 ± 0.02 | 0.52 ± 0.02 | 12.38 ± 0.68 | 11.03 ± 0.70 |
| M.1 2313 | 15.38 ± 0.28 | 76.63 ± 3.52 | 0.87 ± 0.00 | 0.45 ± 0.01 | 6.92 ± 0.22 | 6.05 ± 0.17 |
| M.2 9689 | 13.61 ± 0.18 | 78.87 ± 5.46 | 0.82 ± 0.02 | 0.45 ± 0.00 | 6.21 ± 0.10 | 5.20 ± 0.30 |
| KH | 15.09 ± 0.59 | 114.06 ± 15.02 | 1.21 ± 0.38 | 0.44 ± 0.01 | 6.59 ± 0.23 | 8.80 ± 3.29 |
| F9 | 12.84 ± 0.14 | 98.87 ± 6.29 | 0.78 ± 0.03 | 0.38 ± 0.01 | 4.86 ± 0.08 | 3.79 ± 0.20 |
| DM37 | 13.67 ± 0.30 | 101.78 ± 10.01 | 0.83 ± 0.02 | 0.38 ± 0.02 | 5.15 ± 0.32 | 4.29 ± 0.18 |
| KDSK | 23.00 ± 0.26 | 156.59 ± 36.18 | 0.80 ± 0.04 | 0.43 ± 0.01 | 10.02 ± 0.22 | 8.00 ± 0.42 |
| RBR#04 | 24.4 ± 0.49 | 110.25 ± 19.06 | 0.83 ± 0.05 | 0.49 ± 0.00 | 12.00 ± 0.17 | 10.08 ± 0.48 |
| KNLP | 16.33 ± 0.42 | 77.55 ± 5.65 | 0.82 ± 0.06 | 0.41 ± 0.00 | 6.59 ± 0.14 | 5.50 ± 0.46 |
| DCHMP | 22.43 ± 1.21 | 75.75 ± 8.27 | 0.83 ± 0.01 | 0.44 ± 0.02 | 9.93 ± 0.88 | 8.29 ± 0.68 |
| NMLD | 17.80 ± 0.00 | 149.94 ± 11.24 | 0.85 ± 0.04 | 0.47 ± 0.00 | 8.41 ± 0.05 | 7.13 ± 0.36 |

Results are the means ± SE Values with different letters in the same column are significantly different with P < 0.05. The texture analyser instrumental parameters were derived from Texture analyser (TA-XT plus, Stable Micro System, Godalming, UK) with two-cycle compression test.

**Supplementary Table S5.** KASP and PCR based marker genotyping results.

| Varieties | Wx | SSIIa | 23bp dup |
| --- | --- | --- | --- |
| M1-MT2 | G | GC | - |
| B30-G5 | G | TT | - |
| B30-D4 | G | TT | - |
| PK+5#2E3 | G | TT | - |
| B30-E4 | G | TT | - |
| B30-D5 | G | TT | - |
| RBR#01 | G | TT | - |
| RBR#05 | G | TT | - |
| KHN | T | TT | - |
| PK+5#6E1 | T | TT | - |
| M.1 5678 | T | TT | - |
| PK+6#16F35 | T | TT | - |
| B29-A5 | T | TT | - |
| B11-H5 | T | TT | - |
| PK+6#11F09 | T | TT | - |
| HDSKT 2 | T | TT | - |
| B29-G7 | T | TT | - |
| M.2 10589 | T | TT | - |
| PK+6#3G06 | T | TT | - |
| RBB-005 | T | TT | - |
| RB | T | TT | - |
| JHN | T | TT | - |
| M.2 10853 | T | TT | - |
| M.2 2232 | T | TT | - |
| B11-B11 | T | TT | - |
| RBR#02 | T | TT | dup |
| M.1 2313 | T | GC | dup |
| M.2 9689 | T | GC | dup |
| KH | T | GC | dup |
| F9 | T | TT | dup |
| DM37 | T | TT | dup |
| KDSK | T | TT | dup |
| RBR#04 | T | TT | dup |
| KNLP | T | TT | dup |
| DCHMP | T | TT | dup |
| NMLD | T | TT | dup |

**Supplementary Table S5.** Multivariate statistical models, including path coefficient analysis, confirmatory factor analysis and structural equation models using the Lavaan package in R to estimate various.

| **Statistic determination** | **HRD** | **ADH** | **SPR** | **COH** | **GUM** | **CHEW** |
| --- | --- | --- | --- | --- | --- | --- |
| Comparative Fit Index (CFI) (fit>0.90) | 1.00 | 1.00 | 1.00 | 1.00 | 1.00 | 1.00 |
| Tucker-Lewis Index (TLI) (fit>0.95) | 1.00 | 1.00 | 1.00 | 1.00 | 1.00 | 1.00 |
| RMSEA (Fit<0.08) | 0.00 | 0.00 | 0.00 | 0.00 | 0.00 | 0.00 |
| SRMR (fit<0.08) | 0.00 | 0.00 | 0.00 | 0.00 | 0.00 | 0.00 |
| R^2 (R-square) | 0.66 | 0.73 | 0.40 | 0.38 | 0.58 | 0.58 |

**#Hardness**

**Supplementary Table S6.** Direct (bold diagonal) and indirect effect path coefficients compared with pairwise correlation of HRD.

|  | **AC/AP** | **Protein** | **IDF** | **DFI** | **Pearson’s Correlation with HRD** |
| --- | --- | --- | --- | --- | --- |
| **AC/AP** | **0.323** | -0.093 | 0.139 | 0.162 | 0.531*** |
| **Protein** | -0.088 | **0.343** | -0.084 | 0.078 | 0.248 |
| **IDF** | -0.168 | 0.108 | **-0.267** | 0.021 | -0.307 |
| **DFI** | -0.105 | -0.053 | 0.011 | **-0.499** | -0.647*** |
| **R = 0.339** |  |  |  |  |  |

(*), (**), and (***) indicate statistically significant correlation at p ≤ 0.05, p < 0.01, and p < 0.001. R = residual effect (difference between the model implied covariance matrix and the sample covariance matrix). HRD = Hardness, AC/AP = Amylose content/Amylopectin, Protein = Protein content, IDF = insoluble dietary fibre and DFI = soluble/insoluble dietary fibre.

**Supplementary Table S7.** Statistics summarisation of HRD using the Lavaan package in R.

| **Methods** | **Combinations** | | **Estimate** | **Std.Err** | **z-value** | **P(>\|z\|)** | **Std.lv** | **Std.all** |
| --- | --- | --- | --- | --- | --- | --- | --- | --- |
| **Regressions:** | **HRD ~** | **AC/AP** | 6.659 | 2.629 | 2.533 | 0.011 | 6.659 | 0.323 |
|  |  | **Protein** | 0.864 | 0.267 | 3.237 | 0.001 | 0.864 | 0.343 |
|  |  | **IDF** | -1.492 | 0.666 | -2.240 | 0.025 | -1.492 | -0.267 |
|  |  | **DFI** | -15.983 | 3.492 | -4.577 | 0.000 | -15.983 | -0.499 |
| **Covariances:** | **AC/AP ~~** | **Protein** | -0.062 | 0.039 | -1.579 | 0.114 | -0.062 | -0.273 |
|  |  | **IDF** | -0.053 | 0.019 | -2.774 | 0.006 | -0.053 | -0.521 |
|  |  | **DFI** | -0.006 | 0.003 | -1.856 | 0.063 | -0.006 | -0.325 |
|  | **Protein ~~** | **IDF** | 0.264 | 0.147 | 1.801 | 0.072 | 0.264 | 0.315 |
|  |  | **DFI** | -0.023 | 0.025 | -0.924 | 0.355 | -0.023 | -0.156 |
|  | **IDF ~~** | **DFI** | -0.003 | 0.011 | -0.248 | 0.804 | -0.003 | -0.041 |
| **Variances:** | **HRD** |  | 4.007 | 0.945 | 4.243 | 0.000 | 4.007 | 0.339 |
|  | **AC/AP** |  | 0.028 | 0.007 | 4.243 | 0.000 | 0.028 | 1.000 |
|  | **Protein** |  | 1.862 | 0.439 | 4.243 | 0.000 | 1.862 | 1.000 |
|  | **IDF** |  | 0.378 | 0.089 | 4.243 | 0.000 | 0.378 | 1.000 |
|  | **DFI** |  | 0.012 | 0.003 | 4.243 | 0.000 | 0.012 | 1.000 |

**#Adhesiveness**

**Supplementary Table S8.** Direct (bold diagonal) and indirect effect path coefficients compared with pairwise correlation of ADH.

|  | **AC/AP** | **Protein** | **DFI** | **Pearson's correlation with ADH** |
| --- | --- | --- | --- | --- |
| **AC/AP** | **-0.778** | -0.068 | 0.060 | -0.787*** |
| **Protein** | 0.212 | **0.251** | 0.029 | 0.492** |
| **DFI** | 0.253 | -0.039 | **-0.184** | 0.030 |
| **R = 0.270** |  |  |  |  |

(*), (**), and (***) indicate statistically significant correlation at p ≤ 0.05, p < 0.01, and p < 0.001. R = residual effect (difference between the model implied covariance matrix and the sample covariance matrix). ADH = Adhesiveness, AC/AP = Amylose content/Amylopectin, Protein = Protein content and DFI = soluble/insoluble dietary fibre.

**Supplementary Table S9.** Statistics summarisation of ADH using the Lavaan package in R.

| **Methods** | **Combinations** | | **Estimate** | **Std.Err** | **z-value** | **P(>\|z\|)** | **Std.lv** | **Std.all** |
| --- | --- | --- | --- | --- | --- | --- | --- | --- |
| **Regressions:** | **ADH ~** | **AC/AP** | -205.688 | 25.825 | -7.965 | 0.000 | -205.688 | -0.778 |
|  |  | **Protein** | 8.103 | 3.021 | 2.682 | 0.007 | 8.103 | 0.251 |
|  |  | **DFI** | -75.361 | 39.021 | -1.931 | 0.053 | -75.361 | -0.184 |
| **Covariances:** | **AC/AP ~~** | **Protein** | -0.062 | 0.039 | -1.579 | 0.114 | -0.062 | -0.273 |
|  |  | **DFI** | -0.006 | 0.003 | -1.856 | 0.063 | -0.006 | -0.325 |
|  | **Protein ~~** | **DFI** | -0.023 | 0.025 | -0.924 | 0.355 | -0.023 | -0.156 |
| **Variances:** | **ADH** |  | 525.304 | 123.815 | 4.243 | 0.000 | 525.304 | 0.270 |
|  | **AC/AP** |  | 0.028 | 0.007 | 4.243 | 0.000 | 0.028 | 1.000 |
|  | **Protein** |  | 1.862 | 0.439 | 4.243 | 0.000 | 1.862 | 1.000 |
|  | **DFI** |  | 0.012 | 0.003 | 4.243 | 0.000 | 0.012 | 1.000 |

**#Springiness**

**Supplementary Table S10.** Direct (bold diagonal) and indirect effect path coefficients compared with pairwise correlation of SPR.

|  | **AC** | **AC/AP** | **IDF** | **Pearson's correlation with SPR** |
| --- | --- | --- | --- | --- |
| **AC** | **-3.744** | 3.923 | -0.205 | -0.026 |
| **AC/AP** | -3.718 | **3.950** | -0.192 | 0.04 |
| **IDF** | 2.084 | -2.059 | **0.368** | 0.393* |
| **R = 0.602** |  |  |  |  |

(*), (**), and (***) indicate statistically significant correlation at p ≤ 0.05, p < 0.01, and p < 0.001. R = residual effect (difference between the model implied covariance matrix and the sample covariance matrix). SPR = Springiness, AC = Amylose content, AC/AP = Amylose content/Amylopectin and DFI = soluble/insoluble dietary fibre.

**Supplementary Table S11.** Statistics summarisation of SPR using the Lavaan package in R.

| **Methods** | **Combinations** | | **Estimate** | **Std.Err** | **z-value** | **P(>\|z\|)** | **Std.lv** | **Std.all** |
| --- | --- | --- | --- | --- | --- | --- | --- | --- |
| **Regressions:** | **SPR ~** | **AC** | -0.063 | 0.020 | -3.097 | 0.002 | -0.063 | -3.744 |
|  |  | **AC/AP** | 2.940 | 0.876 | 3.357 | 0.001 | 2.940 | 3.950 |
|  |  | **IDF** | 0.074 | 0.033 | 2.231 | 0.026 | 0.074 | 0.368 |
| **Covariances:** | **AC ~~** | **AC/AP** | 1.226 | 0.290 | 4.228 | 0.000 | 1.226 | 0.993 |
|  |  | **IDF** | -2.535 | 0.869 | -2.919 | 0.004 | -2.535 | -0.557 |
|  | **AC/AP ~~** | **IDF** | -0.053 | 0.019 | -2.774 | 0.006 | -0.053 | -0.521 |
| **Variances:** | **SPR** |  | 0.009 | 0.002 | 4.243 | 0.000 | 0.009 | 0.603 |
|  | **AC** |  | 54.769 | 12.909 | 4.243 | 0.000 | 54.769 | 1.000 |
|  | **AC/AP** |  | 0.028 | 0.007 | 4.243 | 0.000 | 0.028 | 1.000 |
|  | **IDF** |  | 0.378 | 0.089 | 4.243 | 0.000 | 0.378 | 1.000 |

**#Cohesiveness**

**Supplementary Table S12.** Direct (bold diagonal) and indirect effect path coefficients compared with pairwise correlation of COH.

|  | **Ash** | **DFI** | **Pearson's correlation with COH** |
| --- | --- | --- | --- |
| **Ash** | **-0.271** | -0.060 | -0.331* |
| **DFI** | -0.031 | **-0.521** | -0.552*** |
| **R = 0.623** |  |  |  |

(*), (**), and (***) indicate statistically significant correlation at p ≤ 0.05, p < 0.01, and p < 0.001. R = residual effect (difference between the model implied covariance matrix and the sample covariance matrix). Ash = Ash fraction and DFI = soluble/insoluble dietary fibre.

**Supplementary Table S13.** Statistics summarisation of COH using the Lavaan package in R.

| **Methods** | **Combinations** | | **Estimate** | **Std.Err** | **z-value** | **P(>\|z\|)** | **Std.lv** | **Std.all** |
| --- | --- | --- | --- | --- | --- | --- | --- | --- |
| **Regressions:** | **COH ~** | **Ash** | -0.072 | 0.035 | -2.047 | 0.041 | -0.072 | -0.271 |
|  |  | **DFI** | -0.188 | 0.048 | -3.933 | 0.000 | -0.188 | -0.521 |
| **Covariances:** | **Ash ~~** | **DFI** | 0.002 | 0.003 | 0.682 | 0.495 | 0.002 | 0.114 |
| **Variances:** | **COH** |  | 0.001 | 0.000 | 4.243 | 0.000 | 0.001 | 0.623 |
|  | **Ash** |  | 0.021 | 0.005 | 4.243 | 0.000 | 0.021 | 1.000 |
|  | **DFI** |  | 0.012 | 0.003 | 4.243 | 0.000 | 0.012 | 1.000 |

**#Gumminess**

**Supplementary Table S14.** Direct (bold diagonal) and indirect effect path coefficients compared with pairwise correlation of GUM.

|  | **AC** | **Protein** | **DFI** | **Pearson's correlation with GUM** |
| --- | --- | --- | --- | --- |
| **AC** | **0.403** | -0.089 | 0.145 | 0.459** |
| **Protein** | -0.130 | **0.277** | 0.079 | -0.311 |
| **DFI** | -0.116 | -0.043 | **-0.505** | -0.664*** |
| **R = 0.417** |  |  |  |  |

(*), (**), and (***) indicate statistically significant correlation at p ≤ 0.05, p < 0.01, and p < 0.001. R = residual effect (difference between the model implied covariance matrix and the sample covariance matrix). GUM = Gumminess, AC = Amylose content, protein = Protein content, and DFI = soluble/insoluble dietary fibre.

**Supplementary Table S15.** Statistics summarisation of GUM using the Lavaan package in R.

| **Methods** | **Combinations** | | **Estimate** | **Std.Err** | **z-value** | **P(>\|z\|)** | **Std.lv** | **Std.all** |
| --- | --- | --- | --- | --- | --- | --- | --- | --- |
| **Regressions:** | **GUM ~** | **AC** | 0.117 | 0.035 | 3.306 | 0.001 | 0.117 | 0.403 |
|  |  | **Protein** | 0.434 | 0.186 | 2.338 | 0.019 | 0.434 | 0.277 |
|  |  | **DFI** | -10.055 | 2.329 | -4.318 | 0.000 | -10.055 | -0.505 |
| **Covariances:** | **AC ~~** | **Protein** | -3.256 | 1.769 | -1.841 | 0.066 | -3.256 | -0.322 |
|  |  | **DFI** | -0.229 | 0.138 | -1.657 | 0.098 | -0.229 | -0.287 |
|  | **Protein ~~** | **DFI** | -0.023 | 0.025 | -0.924 | 0.355 | -0.023 | -0.156 |
| **Variances:** | **GUM** |  | 1.913 | 0.451 | 4.243 | 0.000 | 1.913 | 0.417 |
|  | **AC** |  | 54.769 | 12.909 | 4.243 | 0.000 | 54.769 | 1.000 |
|  | **Protein** |  | 1.862 | 0.439 | 4.243 | 0.000 | 1.862 | 1.000 |
|  | **DFI** |  | 0.012 | 0.003 | 4.243 | 0.000 | 0.012 | 1.000 |

**#Chewiness**

**Supplementary table S16.** Direct (bold diagonal) and indirect effect path coefficients compared with pairwise correlation of CHEW.

|  | **AC** | **AC/AP** | **DFI** | **Pearson's correlation with CHEW** |
| --- | --- | --- | --- | --- |
| **AC** | **-3.209** | 3.423 | 0.127 | 0.342* |
| **AC/AP** | -3.187 | **3.447** | 0.144 | 0.404* |
| **DFI** | 0.922 | -1.121 | **-0.443** | -0.642*** |
| **R = 0.420** |  |  |  |  |

(*), (**), and (***) indicate statistically significant correlation at p ≤ 0.05, p < 0.01, and p < 0.001. R = residual effect (difference between the model implied covariance matrix and the sample covariance matrix). CHEW = Chewiness, AC = Amylose content, AC/AP = Amylose content/Amylopectin, and DFI = soluble/insoluble dietary fibre.

**Supplementary Table S17.** Statistics summarisation of CHEW using the Lavaan package in R.

| **Methods** | **Combinations** | | **Estimate** | **Std.Err** | **z-value** | **P(>\|z\|)** | **Std.lv** | **Std.all** |
| --- | --- | --- | --- | --- | --- | --- | --- | --- |
| **Regressions:** | **CHEW ~** | **AC** | -0.932 | 0.285 | -3.268 | 0.001 | -0.932 | -3.209 |
|  |  | **AC/AP** | 44.422 | 12.819 | 3.465 | 0.001 | 44.422 | 3.447 |
|  |  | **DFI** | -8.849 | 2.417 | -3.662 | 0.000 | -8.849 | -0.443 |
| **Covariances:** | **AC ~~** | **AC/AP** | 1.226 | 0.290 | 4.228 | 0.000 | 1.226 | 0.993 |
|  |  | **DFI** | -0.229 | 0.138 | -1.657 | 0.098 | -0.229 | -0.287 |
|  | **AC/AP ~~** | **DFI** | -0.006 | 0.003 | -1.856 | 0.063 | -0.006 | -0.325 |
| **Variances:** | **CHEW** |  | 1.942 | 0.458 | 4.243 | 0.000 | 1.942 | 0.421 |
|  | **AC** |  | 54.769 | 12.909 | 4.243 | 0.000 | 54.769 | 1.000 |
|  | **AC/AP** |  | 0.028 | 0.007 | 4.243 | 0.000 | 0.028 | 1.000 |
|  | **DFI** |  | 0.012 | 0.003 | 4.243 | 0.000 | 0.012 | 1.000 |
